# Impact of Body Mass Index and Anthropometric Measures on Cardiorespiratory Fitness in Non-obese Adult Males: A Cross-Sectional Observational Study

**DOI:** 10.7759/cureus.75329

**Published:** 2024-12-08

**Authors:** Kabir Alam, Tribhuwan Kumar, Kamlesh Jha, Md. Zabihullah

**Affiliations:** 1 Physiology, Indira Gandhi Institute of Medical Sciences (IGIMS), Patna, IND; 2 Physiology, All India Institute of Medical Sciences, Patna, Patna, IND

**Keywords:** body mass index: bmi, cardiorespiratory fitness, met (metabolic equivalent), vo2 max, waist circumfernce, waist-to-hip ratio

## Abstract

Introduction

Cardiorespiratory fitness (CRF) is a key health indicator for assessing optimal physical function and overall well-being. Exploring the early impact of body mass index (BMI) and anthropometric measures on CRF in non-obese individuals is essential for identifying risk factors and guiding preventive strategies to address weight-related health challenges. This study aims to investigate the impact of BMI and anthropometric measures on CRF, focusing on maximal oxygen uptake (VO2max) and metabolic equivalents (METs) in non-obese adult males.

Methods

This cross-sectional study included 108 non-obese male participants aged 18-40 years, categorized by BMI into three groups: underweight, normal weight, and overweight. Anthropometric assessments, including height, weight, waist circumference, hip circumference, and skinfold thickness, were conducted. CRF was evaluated by measuring VO2max and METs. Statistical analysis was performed using one-way analysis of variance (ANOVA) for between-group comparisons, followed by Tukey’s post hoc test for pairwise analysis and Pearson’s correlation to examine associations between anthropometric measures and fitness parameters.

Results

Significant differences in VO2max (p < 0.001) and METs (p = 0.013) were found across the BMI categories. Fair negative correlations were observed between BMI and both VO2max (r = -0.382, p < 0.001) and METs (r = -0.384, p < 0.001). Additionally, waist circumference and waist-to-hip ratio showed significant poor negative correlations with these fitness measures, while body density exhibited a fair positive correlation with VO2max (r = 0.37, p < 0.001).

Conclusion

The findings suggest that higher BMI is associated with reduced CRF, as indicated by lower VO2max and MET values, even within the non-obese category. Additionally, waist circumference, waist-to-hip ratio, and body density significantly influence these measures. These results highlight the importance of incorporating these crucial anthropometric factors into health strategies aimed at improving CRF in those with higher BMI, regardless of obesity status.

## Introduction

Cardiorespiratory fitness (CRF), defined as the ability of the circulatory and respiratory systems to deliver oxygen to skeletal muscles for energy production during physical activity, is an essential marker of health [1,2]. Low CRF is strongly associated with an increased risk of cardiovascular disease and all-cause mortality, making it a vital metric for assessing overall physical health [1].

The World Health Organization (WHO) identifies maximal oxygen uptake (VO2max) as the gold standard for measuring CRF [3]. It represents the maximal oxygen flow from the lungs, carried by the circulatory system to the mitochondria within the exercising muscle tissues [4]. VO2max can be estimated through various tests, using either direct or indirect methods. Predictive equations are commonly employed for VO2max estimation due to their cost-effectiveness and relative ease compared to direct measurement [5]. Alongside VO2max, energy expenditure is another vital measure, quantified in metabolic equivalents (METs), where one MET equals 3.5 ml O₂/kg/min, the rate of oxygen consumption at rest [6].

Body mass index (BMI), a widely used measure for categorizing body weight, has been shown in multiple studies to have an inverse relationship with VO2max, due to the negative impact of excess adiposity on oxygen delivery and utilization [4,7]. However, BMI does not distinguish between fat and lean mass and fails to account for variations in body fat distribution, which can vary substantially within a narrow range of BMI, limiting its accuracy as a predictor of CRF [8,9]. Lean mass positively influences oxygen uptake [8], while excess fat, especially visceral adiposity, impairs VO2max through inflammation, insulin resistance, and cardiovascular dysfunction [10].

Body density, which refers to the overall density of the body based on the relationship between fat mass and fat-free mass, provides a more nuanced measure of the balance between these two components [11]. Along with measures of body fat distribution such as waist circumference and waist-to-hip ratio, it offers deeper insights into the balance of fat and lean mass and their effects on fitness outcomes [11-13]. However, the interplay of these variables, particularly in non-obese populations, remains inadequately explored, as the majority of studies focus on obesity, often overlooking the impact on CRF in non-obese individuals.

This study investigates the impact of BMI and anthropometric measures on CRF, focusing on VO2max and METs in non-obese adult males. We hypothesize that these measures, even within the non-obese range, will show significant correlations with CRF. By exploring these relationships, this study aims to fill the research gap by providing insights into how BMI and anthropometric factors influence CRF in individuals without obesity.

## Materials and methods

Participant recruitment and eligibility criteria

The study was conducted at All India Institute of Medical Sciences (AIIMS) Patna, Bihar, India. This study included 108 non-obese, apparently healthy adult male participants, aged 18 to 40 years, with a BMI under 30 kg/m². Participants were recruited through outreach efforts targeting the community living in the vicinity of the institute. Those diagnosed with medical conditions that could influence the study parameters, such as cardiovascular, metabolic, or respiratory disorders, were excluded to minimize potential confounding factors related to pre-existing health conditions. Written informed consent was obtained from all participants, in accordance with the study protocol approved by the Institutional Ethics Committee of All India Institute of Medical Sciences (AIIMS) Patna (Ref. No.: AIIMS/Pat/IEC/PGTh/Jan 19/23).

The study was conducted over a 15-month period, with a smaller sample size and a decision to include only male participants to ensure feasibility and consistency, given the logistical constraints of recruitment within the study timeframe. Despite these limitations, the study provides valuable insights into the relationship between BMI, anthropometric measures, and CRF in individuals without obesity. Future studies with larger sample sizes and multicenter collaborations are recommended to validate and expand these findings.

Anthropometric measurements

Height and weight were measured by a single observer following standard protocols to ensure consistency, and BMI was calculated using the Quetelet index [14]:

BMI = body weight (kg) / height (m)^2^

Participants were categorized into three groups based on the WHO BMI classification system [9,15]: underweight (BMI < 18.5 kg/m²), normal weight (BMI 18.5-24.9 kg/m²), and overweight (BMI 25-29.9 kg/m²).

Skinfold thickness was measured at seven sites of the body while the subject stood upright, using a calliper placed directly on the skin surface. Body density was calculated using the Jackson and Pollock formula [16]. Waist and hip circumferences were measured using a stretch-resistant tape according to WHO guidelines [17]. The waist-hip ratio was calculated as the circumference of the waist divided by that of the hip.

VO2max estimation and metabolic equivalent (MET) calculation

The Ebbeling single-stage treadmill test [18] was employed to estimate VO2max and calculate MET values. This test was conducted in the Department of Physiology at AIIMS Patna, using a desktop computer-based treadmill stress test system (Model: Mortara Wireless Acquisition Module WAM-114500239471, Mortara Instrument, Milwaukee, Wisconsin, United States). 

Test Procedure

Warm-up phase: The warm-up phase lasted for four minutes, during which participants were asked to walk at a comfortable speed between 2.0 and 4.5 miles per hour (mph), set on a 0% grade. The goal was to elicit a heart rate response within 50% to 70% of the age-predicted maximum heart rate (MHR). If the heart rate response did not fall within this range by the end of the first minute, the speed was adjusted accordingly.

The age-predicted MHR was calculated for each subject using the formula [19]:

MHR = 208 − (0.7×age)

Test proper: Following the warm-up, participants maintained the same speed for an additional four minutes at a 5% grade. During this period, steady-state heart rate (SS HR) was recorded by averaging the final 30 seconds of the last two minutes at the 5% grade. To confirm steady-state, the heart rate must not fluctuate by more than 5 bpm between the two final minutes. If the variation exceeds 5 bpm, the test duration is extended by one minute, and SS HR is then recorded from the last two minutes. The SS HR is expressed in beats per minute (bpm).

Calculation of VO2max

Post-test, VO2max (mL/(kg·min)) was calculated using the equation [18]:

VO2max = 15.1 +21.8 (speed in mph) - 0.327 (SS HR in bpm) - 0.263 (speed in mph x age in years) + 0.00504 (SS HR in bpm x age in years) + 5.98 (gender; female = 0, male = 1)

MET Calculation 

One MET is defined as the amount of oxygen consumed while sitting at rest and is equal to 3.5 ml O2 per kg body weight x min [20]. MET values were automatically detected by the treadmill software.

Statistical analysis

Statistical analyses were performed using IBM SPSS Statistics for Windows, Version 22 (Released 2013; IBM Corp., Armonk, New York, United States). The Shapiro-Wilk test confirmed the normal distribution of all continuous variables; therefore, values are presented as means ± standard deviations, and parametric tests were applied. Group comparisons across BMI categories (underweight, normal weight, and overweight) were conducted using one-way analysis of variance (ANOVA) tests, followed by Tukey’s post hoc test for pairwise comparisons. Categorical variables, presented as frequencies and percentages, were analyzed using the chi-square test or Fisher’s exact test, as appropriate. Pearson's correlation coefficient was employed to assess associations between anthropometric measures and fitness parameters. The strength of the correlations was evaluated based on the criteria described by Chan et al.: values below 0.3 indicate a poor relationship, 0.3 to 0.5 indicate a fair relationship, 0.6 to 0.8 indicate a moderately strong relationship, and 0.8 and above indicate a very strong relationship [21]. A p-value < 0.05 was considered statistically significant. 

## Results

Demographic characteristics

Out of the total 108 participants, 13 were in the underweight category, 72 were in the normal weight category, and 23 were in the overweight category. The demographic information of the participants is presented in Table 1, showing no statistically significant differences in age, dietary habits, and occupation among the three BMI categories, indicating homogeneity of the study population across these demographic variables. 

**Table 1:** Distribution of participants in terms of demographics. p-values were determined using the one-way ANOVA test or the ^a^Chi-square/Fisher exact test. ANOVA: analysis of variance; BMI: body mass index; SD: standard deviation

Parameter			BMI categories			p-value
			Underweight (n = 13)	Normal weight (n = 72)	Overweight (n = 23)	
Age (year) (mean (SD))			26.69 (7.67)	28.74 (6.30)	31.30 (4.54)	0.08
Dietary habit (frequency (%))	Vegetarian		1 (4.8)	18 (85.7)	2 (9.5)	0.17^a^
	Non-vegetarian		12 (13.8)	54 (62.1)	21 (24.1)	
Occupation (frequency (%))		Unskilled	7 (26.9)	16 (61.5)	3 (11.5)	0.09^a^
		Semiskilled	3 (7.7)	27 (67.5)	10 (25)	
		Skilled	3 (7.1)	29 (69)	10 (23.8)	

Cardiorespiratory fitness measures across body mass index categories

The CRF measures (VO2max and MET) of study subjects of the different BMI categories are shown in Table 2, demonstrating a statistically significant difference in VO2max (p < 0.001) and MET (p = 0.013) among the three BMI categories. Post hoc analysis using Tukey's test shows that there was a significant difference between the CRF parameters (VO2max and MET) of the underweight BMI category and the overweight BMI category, as well as between the normal BMI category and the overweight BMI category. But there was no significant difference between underweight and normal BMI categories. The mean VO2max for each BMI category was as follows: 50.26 mL/(kg·min) for the underweight category, 48.77 mL/(kg·min) for the normal weight category, and 43.59 mL/(kg·min) for the overweight category. The mean MET for each BMI category was as follows: 6.93 for the underweight category, 6.89 for the normal weight category, and 6.11 for the overweight category. These findings highlight the decline in CRF measures with increasing BMI, particularly in the overweight category.

**Table 2:** Comparison of cardiorespiratory fitness measures (VO2max and MET) between different BMI categories. Data are shown as mean (SD). p-values were determined using the one-way ANOVA test. Letters with different superscripts in the same row are significantly (p < 0.05) different according to Tukey's post hoc test. ANOVA: analysis of variance; SD: standard deviation; BMI: body mass index; VO2max: maximal oxygen uptake; MET: metabolic equivalent

Parameter	BMI categories			p-value
	Underweight (n = 13)	Normal weight (n = 72)	Overweight (n = 23)	
VO2max (mL/(kg·min)) (mean (SD))	50.26 (8.43)^a^	48.77 (7.19)^a^	43.59 (9.15)^b^	<0.001
MET (mean (SD))	6.93 (0.69)^a^	6.89 (0.74)^a^	6.11 (0.96)^b^	0.013

Correlation between cardiorespiratory fitness and anthropometric parameters

The estimated Pearson correlation coefficients for CRF measures (VO2max and MET) with body mass index, waist circumference, waist-hip ratio, and body density are shown in Table 3, indicating statistically significant fair negative correlations between BMI and VO2max (r = -0.382, p < 0.001) and between BMI and MET (r = -0.384, p < 0.001) and a fair positive correlation between body density and VO2max (r = 0.37, p < 0.001). These findings suggest that higher BMI is associated with reduced CRF measures, while greater body density, often indicative of higher lean mass, correlates positively with aerobic capacity.

**Table 3:** Correlation of cardiorespiratory fitness measures (VO2max and MET) with body mass index, waist circumference, waist-hip ratio, and body density. ^a^Analyzed by Pearson correlation analysis VO2max: maximal oxygen uptake; MET: metabolic equivalent

Parameter	VO2max		MET	
	Correlation coefficient	p-value^a^	Correlation coefficient	p-value^a^
Body mass index	-0.382	<0.001	-0.384	<0.001
Waist circumference	-.250	0.009	-.259	0.007
Waist-hip ratio	-0.224	0.020	-.217	0.024
Body density	0.37	<0.001	0.27	0.005

## Discussion

The present study investigated the impact of BMI on CRF measures, namely VO2max and METs, in non-obese adult males. Our results revealed statistically significant differences in both VO2max and METs across BMI categories, with post hoc analysis showing a significant decrease in these measures in the overweight category compared to the underweight and normal BMI categories. Pearson correlation analysis demonstrated fair negative correlations between BMI and CRF measures, highlighting the detrimental effect of higher BMI on aerobic capacity. A fair positive correlation was observed between body density and VO2max, indicating that higher lean mass relative to fat mass supports better fitness outcomes. Additionally, poor negative correlations between waist circumference, waist-hip ratio, and both VO2max and METs further highlight the potential influence of central adiposity in restricting CRF, illustrating the intricate interplay of various factors in shaping CRF outcomes. 

VO2max, or maximal oxygen uptake, is a widely accepted marker of aerobic capacity, serving as a gold-standard measure of an individual's overall CRF [1]. Our study’s results, which show a fair negative correlation between BMI and both VO2max and METs, with significant differences in these measures across BMI categories, align with previous studies that report an inverse relationship between BMI and aerobic capacity [4,7,22,23]. For instance, Mohammed et al. [7] linked lower BMI within healthy ranges to optimized VO2max attainment, implying enhanced respiratory efficiency to sustain exercise intensity and duration. The physiological mechanisms behind this relationship suggest that excess body weight, particularly adiposity, impairs the body's ability to deliver and utilize oxygen effectively during physical activity, thus leading to a decrease in VO2max [4,23]. Similarly, METs, which are commonly used to estimate the intensity of physical activity, also differed significantly across BMI categories in our study. This observation is consistent with Bondage B et al. [24], who reported that individuals with higher BMI values generally demonstrate lower METs, suggesting reduced aerobic fitness and a limited capacity to engage in moderate-intensity physical activity.

However, some studies offer a more nuanced perspective, indicating that the relationship between BMI and fitness is not solely determined by body weight. For instance, McInnis and Balady [8] found that bodybuilders with low body fat percentages had significantly higher VO2 during submaximal effort compared to men with normal body fat but similar body mass. This study highlights that muscle mass, rather than body weight alone, plays a pivotal role in determining VO2max. Our study further supports this complexity by demonstrating that higher body density, often reflective of greater lean mass, was associated with better CRF measures, reinforcing the importance of body composition as a determinant of fitness outcomes alongside BMI.

In our study, we also observed statistically significant poor negative correlations between waist circumference, waist-hip ratio, and VO2max and METs. This finding aligns with a large body of literature suggesting that central obesity, particularly visceral fat, plays a key role in reducing CRF [12,13,25]. Moreover, Dagan SS et al. [26] reported significant negative correlations between BMI and VO2max, as well as waist circumference and VO2max, in healthy men, further emphasizing the role of central obesity in limiting aerobic fitness. The accumulation of visceral fat has been linked to mitochondrial dysfunction, increased oxidative stress, greater inflammation, insulin resistance, and cardiovascular dysfunction, all of which impair VO2max [10,27]. Our findings suggest that reducing central obesity could play a crucial role in enhancing VO2max and METs, which are vital for overall health and well-being. Future research should explore these multifaceted relationships across diverse populations to develop effective strategies for improving CRF in individuals with higher BMI, even among those classified as non-obese. 

Limitations

This study has several limitations. The sample size was relatively small and composed solely of non-obese adult males, which may limit the generalizability of the results to other populations, including females or individuals with obesity. Additionally, factors like physical activity or genetics were not considered, and these could influence CRF. Although anthropometric measures were utilized, they may not fully capture body composition, which could provide a more comprehensive understanding of the factors affecting CRF. Additionally, our study employed an indirect estimation method for measuring VO2max, chosen for its feasibility, but direct measurement is considered more accurate. Future research should address these limitations by including larger, more diverse populations; considering factors like physical activity and genetics; using more precise body composition measures; and employing direct VO2max measurements. 

## Conclusions

In conclusion, our study provides valuable insights into the impact of BMI and anthropometric measures on CRF in non-obese adult males. The significant decrease in VO2max and METs in the overweight category compared to underweight and normal BMI categories highlights the detrimental effect of higher BMI on aerobic capacity. Additionally, our findings indicate that higher body density, often indicative of greater lean mass, is associated with improved CRF, highlighting body composition as a critical determinant alongside BMI. Moreover, the statistically significant poor negative correlations between waist circumference, waist-to-hip ratio, and fitness measures further emphasize the potential role of central adiposity in limiting CRF. While larger studies are needed to expand on these findings, our results suggest that improving body composition, particularly increasing lean mass and reducing central adiposity, could be key strategies for enhancing cardiorespiratory fitness, even in non-obese individuals. 
